# Caterpillar Envenomation (Lepidopterism) in a Panamanian Jungle: About a Case

**DOI:** 10.7759/cureus.51247

**Published:** 2023-12-28

**Authors:** Jose A Suarez, Monica R Pachar Flores, Maria E Osorio Marin, Juan C Navarro

**Affiliations:** 1 Faculty of Health Sciences, Universidad Internacional SEK (UISEK), Quito, ECU; 2 Infectious Diseases, Hospital Santo Tomás, Panama, PAN; 3 Infectious Diseases, Instituto Oncologico Nacional, Panama, PAN; 4 General Medicine, Hospital Manuel A. Nieto, Darien, PAN; 5 Entomology, Universidad Internacional SEK (UISEK), Quito, ECU

**Keywords:** megalopyge, toxicology and envenomation, lepidopterism, darien gap, caterpillar envenomation

## Abstract

Caterpillar venom has the potential to cause acute pain and systemic symptoms in individuals seeking medical attention in the jungles of Panama. Although this is not an obligatory notifiable disease, the hazards associated with exposure to this animal are widely recognized within the local community.

Here, we present a case of a patient who sought medical attention after being rescued from a river in a Panamanian jungle after feeling acute pain in an upper extremity associated with shortness of breath and how tropical medicine teleconsult allowed for quick identification of the cause and assisted in the management.

About his case, we examine the phenomenon of caterpillar envenomation and suggest that further research is needed to assess the potential impact of climate change on this disease. Of particular concern is the likelihood of an increase in contact accidents. We recommend that scientists and public health officials work together to understand the mechanisms of this disease better and to develop effective strategies for prevention and treatment. Our analysis underscores the importance of ongoing monitoring and surveillance to ensure we are prepared for future outbreaks.

## Introduction

Acute pain while swimming in a river in the jungle has several differential diagnoses to consider with accidental contact with the local flora and fauna. When systemic symptoms appear immediately after the event, envenomation should be higher in the list of differential diagnoses.

There is currently a lack of understanding regarding accidents involving wild animals in Panama, and further research is needed to determine the actual frequency of these occurrences. These incidents often occur in rural and jungle areas where access to anti-venom therapy is limited, and established treatment protocols still need to be in place.

Contact accidents associated with exposure to water in Panama reflect the biodiversity of the country: reptiles such as snakes (venomous and not venomous), amphibians, and the spectacled caiman. Contact with flying animals, like bats, may happen, although it has not been reported in our country [1,2].

Venomous stings and bites that can happen in the tropics include contact accidents with arthropods (e.g. Arachnida like Phoneutria, Insecta like Vespidae, or Myriapods like millipedes), they may be near the river's edge or errant on the surface [3].

Caterpillar envenomation is one of Panama's many diseases associated with contact accidents with wild animals. It is the product of indirect or direct contact with the hairs of the caterpillar, and it results in a range of clinical manifestations that, in some cases, may be life-threatening.

Insects of the order Lepidoptera are responsible for this envenomation and are arranged in over 100 families with worldwide distribution. Only nine produce human disease; the Saturniidae family harbors the deadliest species: *Lonomia spp* [4,5]. Caterpillar venom cocktail is not as understood as snakes, as well as its epidemiology and the true burden of the envenomation.

Comparing the epidemiology of envenomation by terrestrial venomous animals in Brazil, among 1,192,667 accidents during 12 years, snakes and scorpions are the main ones responsible while caterpillars have been the lowest. Nevertheless, a tendency of steadily increased reports during the study was noted [6]. However, it is important to know that the authors made no distinction between species of caterpillars from 2006-2012, which underestimated the clinical spectrum of this disease.

Other data from Brazil, which reflects only their local epidemiology, shows that the incidence of this envenomation is 3.2 cases per 100,000 habitants, with 33 cases of death among 40,588 cases [4].

In this report, we present a case of a young Amerindian woman (Embera-Wounnan) who encountered an unusual caterpillar while washing her clothes in the wilderness at a river edge and how modern telecommunication through rural medicine teleconsulting with a tropical medicine group in Panama City helped the local medical doctor properly manage a life-threatening condition in a low-resource setting.

## Case presentation

A 16-year-old female without past medical history was admitted to a rural hospital in the Darien Jungle with a history of acute shortness of breath (SOB) and acute burning pain in her left hand while swimming in a river.

Her family took her 15 minutes after the event to a rural hospital near Meteti, Darien. They described that the patient encountered a grayish worm with abundant hairs known by the locals as chicken worm (in Spanish: “gusano pollo”) floating near the river's border that accidentally stung his right hand.

**Figure 1:**
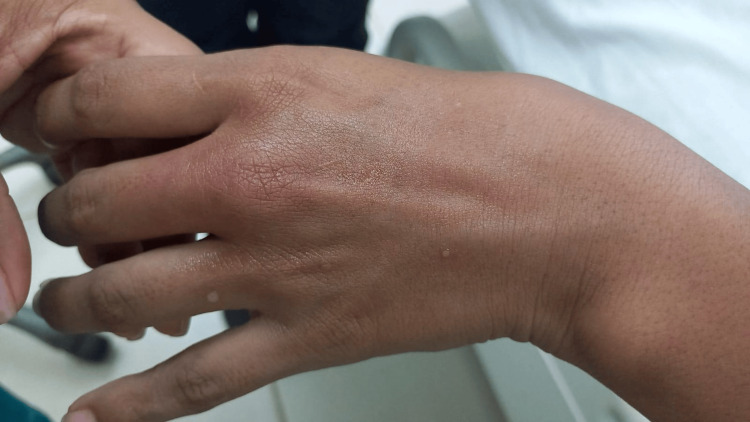
Site of contact in the left hand of the patient Erythema over the third metacarpophalangeal articulation that extends to the dorsal aspect of the hand. The patient refired burning pain and did not tolerate touch.

At the emergency room, she was acutely ill, with chest pain and SOB associated with exquisite burning sensation, tingling, and pins and needles pain (graded 10/10) at the dorsal area of the left hand. At the physical exam, she had BP 100/60 mmHg, HR: 100 bpm, RR 20, and O_2_ saturation at room air: 98%, T: 36,2 °C. The hand had non-pitting painful edema and erythema and was warm to the touch (Figure 1); upon closer examination, no evident spines were seen. However, a scotch tape test was performed, where tiny spines were seen.

She was admitted with a diagnosis of caterpillar-associated envenomation. Tropical medicine advisors from Panama City supported the patient's management at the rural hospital through teleconsulting, guiding management (election of medication and dosing, and planning of hospital transfer and further management at a tertiary level hospital if needed) after classification of the type of caterpillar envenomation. Several images of different caterpillars were shown to the patient, and the identification resulted in *Megalopyge spp*.

No guidelines exist in our country to determine the best management of this patient. However, the support provided by the team of experts in tropical medicine oriented it, who maintained constant communication with the team in Darien. The syndrome was classified as erucism with overlapping lepidopterism because of systemic symptoms.

Since no antivenom exists for this caterpillar, the patient received symptomatic management and was monitored as an inpatient for the development of systemic complications. She received antihistaminic (diphenhydramine 50 mg IV every 8 hours), methylprednisolone 125 mg IV single doses, tramadol 50 mg PO every 8 hours, and pregabalin 75 mg PO every night.

Due to the unavailability of access at the medical facility, bloodwork could not be performed. Nevertheless, this does not impact the planned course of treatment for the patient. After 24 hours of hospital treatment, the patient's symptoms showed signs of improvement, and she was discharged and prescribed oral medications of pregabalin and tramadol for outpatient management.

## Discussion

Caterpillar envenomation can be classified according to the clinical presentation in erucism, lepidopterism, dendrolimiasis, ophtalmia nodosa, and consumptive coagulopathy with secondary fibrinolysis; however, on some occasions, this syndrome may overlap [7]. The clinical difference of these syndromes can be seen in Table 1.

**Table 1:** Caterpillar envenomation syndromes Adapted from [4]

Erucism	Lepidopterism	Dendrolimiasis	Ophtalmia nodosa	Consumptive coagulopathy with secondary fibrinolysis.
Dermatitis caused by contact or airborne exposure to caterpillar irritants.	Systemic illness caused by direct or indirect contact with caterpillar, cocoon, or moth urticating hairs, spines, or body fluids. Symptoms include upper respiratory and gastrointestinal distress, as well as bronchospasm and dyspnea.	A chronic form of lepidopterism caused by direct contact with caterpillars of the Central Asian pine-tree lappet moth (Dendrolimus pini). Symptoms include urticating maculopapular dermatitis, migratory inflammatory polyarthritis, migratory inflammatory polychondritis, chronic osteoarthritis, and rarely, acute scleritis.	Chronic ocular condition characterized by initial conjunctivitis, followed by pan-uveitis caused by the penetration of the cornea and migration of irritating hairs into the eye.	Severe hemorrhagic manifestations. Described in cases of Lonomia spp.

This patient had a clinical syndrome compatible with erucism and overlapping lepidopterism. The clinically relevant species responsible for this disease include Megalopygidae, Arctiidae, Saturnidae, and Hemileucinae [8]. The envenomation can occur through direct contact with the spikes (true direct erucism), indirect contact with the spikes (true indirect erucism), meta-erucism (caused by the persistence of spikes in adult forms), and para-erucism (caused by cryptotoxic caterpillars) [8,9].

Certain types of caterpillar venom can be extremely dangerous, such as the one produced by *Lonomia spp*. Antivenom for this species is available in some countries; however, Panama is one of the exceptions. This venom can sometimes cause intravascular coagulation disorder and bleeding - leading to potentially fatal outcomes. Reports indicate that the mortality rate of these cases is comparable to that of snake bites in some areas of Brazil [8].

The true impact of stings from venomous caterpillars on communities residing in jungles remains unclear, with an unbalance between sightings reported in software such as iNaturalist and the reports of envenomations. In such instances, obtaining proper medical attention is imperative for individuals experiencing symptoms following a bite. Moreover, emergency care may be necessary. Identifying the specific species of caterpillar responsible for the bite is crucial in evaluating the likelihood of complications or potential deaths.

The caterpillar, in our case, is a member of the family *Megalopygidae.* This animal is known by several folkloric names in Latin America associated with the intense neuropathic pain that their sting produces and its appearance: “oruga de fuego” (fire caterpillar), “gusanos flecha” (arrow worm), “gusano pollo” (worm chicken), puss caterpillar, or wooly slug.

According to the Global Biodiversity Information Facility (GBIF) in Panama, there are 137 records corresponding to six species of *Megalopyge (M.)*. The cause of the accident that the patient identified through an image possibly corresponds to *M. opercularis*, of which there is only one record in Panama from 1977 [10]. Since we do not have the sample directly for its exact identification, we assume here that it most likely corresponds to a specimen of *Megalopyge *and is close to *opercularis *(*Megalopyge* near *opercularis*) because the larvae of the other species are phenotypically different.

The Megalopyges caterpillar species, *M. opercularis*, has been the subject of extensive research in Latin America due to its wide distribution. This particular caterpillar boasts a tear-shaped body that ranges from 2.5 to 3.5 cm in length and is covered in fluffy hairs. Its coloration varies from dirty white to gray-yellow or brown. The species is characterized by two types of hairs: true venomous spikes and longer, harmless hairs resembling regular hairs [7-9,11].

Once the individual is in contact with the animal, immediate local symptoms develop secondary to the venom and, in some cases, systemic. Immediately after the sting, the site develops a distinct lesion surrounded by an erythematous halo with an inner footprint or grind-like pattern reflecting each broken-spine hypodermic injection point. The site may become hemorrhagic and present with bulla or pustules [7].

The most important clinical manifestation of erucism by *Megalopygidae* is pain, which can be manifested as radicular pain. Previous authors have used a pain scale to better understand the severity of the pain with the Numeric Pain Rating Scale (NPRS), to decide the proper treatment that can guide management [12].

Other symptoms compilated by the Texas poison centers of the United States, from 2001-2016 were other dermal manifestations (irritation, erythema, and edema) and systemic symptoms: gastrointestinal (nausea, vomits), neurologic (numbness), cardiovascular (chest pain), ocular, and respiratory [13].

Based on literature reports, experiences, and guidelines from poison centers in Brazil and the USA, our tropical medicine consultant team recommended using antihistamines, steroids, and pain relievers. Although there is no specific antivenom for *Megalopyge spp.*, a specific antivenom is available for another caterpillar species: *Lonomia spp*. This was produced in the Butantan Institute as a response to an outbreak in Brazil in 1989 [4], where national guidelines are also used as a reference in other countries in Latin America.

Recent evidence suggests that the current climatic conditions of warmer temperatures cause changes in the life cycles of the caterpillars. Awareness of these envenomations is needed as more cases are expected due to climate change [11,14,15].

## Conclusions

Even though this is the first case report of erucism in Panama, this is a known disease among the population across the country, with an important oral tradition of known danger if touched. The increased number of publications and general interest in the subject reflects the anthropogenic change in the environment that we are experiencing as a consequence of climate change, where we may expect an increase in the report of this type of accident as urban areas expand to the natural niche of *Megalopyge spp.*

Effective management and heightened suspicion are crucial in handling worst-case scenarios. In our case, swift identification of the caterpillar was made possible through sharing photos via a cell phone online connection. This provided appropriate management and reassurance to the local medical professionals regarding what to expect and when to call for airborne transportation to a third-level hospital. These actions contributed to the positive outcome for our patient.

## References

[1] Birding W. Panama Wildlife (2020). Panama wildlife: a glimpse at great biodiversity. https://www.whitehawkbirding.com/panama-wildlife-a-glimpse-at-great-biodiversity/.

[2] Dato VM, Campagnolo ER, Long J, Rupprecht CE (2016). A systematic review of human bat rabies virus variant cases: evaluating unprotected physical contact with claws and teeth in support of accurate risk assessments. PLoS One.

[3] Xin Y, Arief IDJ, Shajahan R, Arief FJ, Pillai N (2021). Venomous stings and bites in the tropics (Malaysia): review (non-snake related). Open Access Library Journal.

[4] Seldeslachts A, Peigneur S, Tytgat J (2020). Caterpillar venom: a health hazard of the 21st century. Biomedicines.

[5] Favalesso MM, Lorini LM, Peichoto ME, Guimarães AT (2019). Potential distribution and ecological conditions of Lonomia obliqua Walker 1855 (Saturniidae: Hemileucinae) in Brazil. Acta Trop.

[6] Chippaux JP (2015). Epidemiology of envenomations by terrestrial venomous animals in Brazil based on case reporting: from obvious facts to contingencies. J Venom Anim Toxins Incl Trop Dis.

[7] Diaz JH (2005). The evolving global epidemiology, syndromic classification, management, and prevention of caterpillar envenoming. Am J Trop Med Hyg.

[8] Juan P. Gómez C (2014). Lepidopterism and erucism in Colombia. Biosalud.

[9] Alvaro Delgado HP (1966). Lepidopterismo y erucismo. Epidemiologia y aspectos clinicos en el Peru [Article in Spanish]. Mem Inst Butantan Simp Internac.

[10] (1977). Megalopyge opercularis (J.E.Smith, 1797) [In Spanish]. https://www.gbif.org/es/occurrence/2563034074.

[11] (2023). FUNASA. Manual de Diagnostico e Tratamento de Acidentes por Animais Peonhentos [In Website]. https://www.icict.fiocruz.br/sites/www.icict.fiocruz.br/files/Manual-de-Diagnostico-e-Tratamento-de-Acidentes-por-Animais-Pe--onhentos.pdf..

[12] Branco MM, Borrasca-Fernandes CF, Prado CC (2019). Management of severe pain after dermal contact with caterpillars (erucism): a prospective case series. Clin Toxicol (Phila).

[13] Forrester MB (2018). Megalopyge opercularis caterpillar stings reported to Texas poison centers. Wilderness Environ Med.

[14] Hall P. Caterpillars Provide New Clues On Impact of Warmer Temperatures [WebPage]. 2012 [updated (2023). Caterpillars provide new clues on impact of warmer temperatures. https://columbian.gwu.edu/caterpillars-provide-new-clues-impact-warmer-temperatures.

[15] Hood GR, Comerford M, Weaver AK, Morton PM, Egan SP (2019). Human-mediated disturbance in multitrophic interactions results in outbreak levels of North America's most venomous caterpillar. Biol Lett.

